# Annotations

**Published:** 1895-06-01

**Authors:** 


### ANNOTATIONS.

### The Midwives Bill.

Dr. Rentoul, of Liverpool, has sent to us some "opinions of counsel" concerning the Midwives Registration Bill now before Parliament. Undoubtedly Dr. Rentoul has earned the gratitude of the medical profession and the public for so thoroughly sifting the proposals contained in the new Bill, and so clearly showing wherein some of them require to be modified, amended, strengthened, or deleted. We cannot agree that the Bill as it stands bristles with the dangers which spring up in such fertile crops in the imagination of Dr. Rentoul and other practitioners. On the contrary, we must insist that it is well devised to create an order of midwives who, if the medical men constituting the proposed Midwifery Board do their duty, can never be anything but midwives or "midwifery nurses," if that term be preferred. Dr. Rentoul raises ten thousand ghosts of possible dangers. The one and all-sufficient exorciser of these ghosts will be the Midwifery Board which the new Bill proposes to create, and which will consist entirely of medical men selected by competent bodies for their special and individual competence. But notwithstanding our firm trust in the Midwifery Board, we entirely agree with Dr. Rentoul that the Bill itself, before it becomes law, should, in its details as well as in its general principles, provide the most perfect safeguards which can be devised for the protection of medical education and the maintenance of the present high standards of examination and qualification; and should also be strengthened by all needful penalties against the infringement by midwives of the Medical Acts in any single particular. Our position is this, and we think it ought to be the position of every medical practitioner, that the present unlicensed practice of midwifery is a scandal and a danger to the community; that the scandal and danger can be removed by the training and registration of all persons practising midwifery; that the present Bill should be so modified as to render such training and registration compulsory on all those who in future undertake the work of attending women in their confinements; and that if it be adequately discussed and amended in committee, it may be rendered worthy of the support of the community and the whole medical profession.

### Doctor or Spy?

Every year new duties are thrown on the medical profession, and it is difficult even to guess what will not ultimately be included within the manifold functions of the doctor. But at least we may hope that the day is far distant when it will be considered a proper and seemly thing to ask one medical man to act as spy upon another. And yet, according to a published letter signed by Dr. Waldo, that is very much what has been done by the Vestry of St. George's, Southwark, in sending their medical officer to visit such of their employés as send in medical certificates of sickness. It is, perhaps, as well never to be surprised at anything that happens in the municipal management of London; but certainly this action of the vestry shows a more than ordinary lack of perception of the proper function of a health officer. If there is one thing more important than another for the smooth working of the Public Health Act, it is that the medical officer of health should be on good terms with the doctors in his district; and, although it is true that the specialisation of sanitary work is one great reason for the provision, now common in most large towns, that the medical officer shall not engage in private practice, still, one of the great advantages of this restriction is that it has been found by experience to conduce greatly to those friendly relations between him and his professional brethren which are found to be so necessary to sanitary administration. And yet it is proposed to upset these relations and introduce friction in every direction by sending the medical officer to make what Dr. Waldo speaks of with indignation as "surprise visits" on the patients of other medical men, without consultation or previous communication with them. It is perfectly open to a vestry, like any other employer of labour, to provide medical attendance for its workpeople (but in that case general practitioners should be employed, not sanitary specialists); it is also open to them, although it is a hardship on their workpeople, to demand a consultation where illness is prolonged beyond a certain period; or they may do as every club and benefit society in the country does, viz., accept the certificate of the medical man in attendance; but it certainly is not open to them to set one doctor to act as spy upon another.

### The Birmingham Hospital Saturday Fund.

The Birmingham Hospital Saturday Fund returns amounted to £9,546 on the first day. So far the collection is larger than any previous year, but the point for notice is that the amounts given are considerably larger per contribution than on any previous year since 1885. The number of contributors up to nine o'clock on Saturday evening was returned as 809 in 1885, and the offerings amounted to £5,095. In 1895 the number of contributions is stated to have been 995 on Saturday night, yielding no less than £9,546, or nearly an average of £10 per collection. On Monday, the sums paid in increased the total receipts to nearly £11,000, and there is every prospect that the £13,000 which the Committee have asked for this year will be forthcoming. It is instructive to inquire how these exceptionally favourable results have been secured? They afford evidence of the truth of our oft repeated assertion, that the working classes, if their interest can be aroused, will cheerfully give all the money to hospitals which can be justly demanded from them. The secret of the Birmingham success lies in the fact that a few men have devoted their whole energies to the work. No opportunity has been lost to impress the working classes of Birmingham with the feeling that not only the movement itself but the maintenance of the convalescent homes for men and for women, and everything connected with the Fund, depend upon their efforts and support. Mr. Smedley, the secretary, is indefatigable. Every fortnight he goes down to Llandudno to photograph the patients who have been resident at the convalescent institutions. These photographs are treasured by the patients, and copies of them are now to be met with in the majority of artizans' homes. Everywhere and in everything this central fact of the responsibility of the working classes for the due maintenance of the Hospital Saturday Fund is kept well to the fore. In consequence, the movement is widely popular in Birmingham. Every working man and woman is proud of the Fund, and its work and welfare are regarded as of paramount importance to the whole population of the Midland metropolis. We heartily congratulate Alderman Cook, Mr. Smedley, and all who have helped to achieve this notable success. It is one which should put heart into the working classes everywhere, and tend to strengthen and make permanent the voluntary system of hospital support in every part of the country.

